# Supplementary material for: Endothelial Cell Response to Combined Photon or Proton Irradiation with Doxorubicin
Source: Int J Mol Sci. 2023 Aug 16;24(16):12833. doi: 10.3390/ijms241612833 (PMC10454477; doi:10.3390/ijms241612833)
Supplement: Supplementary file 1 [file ijms-24-12833-s001.zip › ijms-2509285-supplementary.pdf]

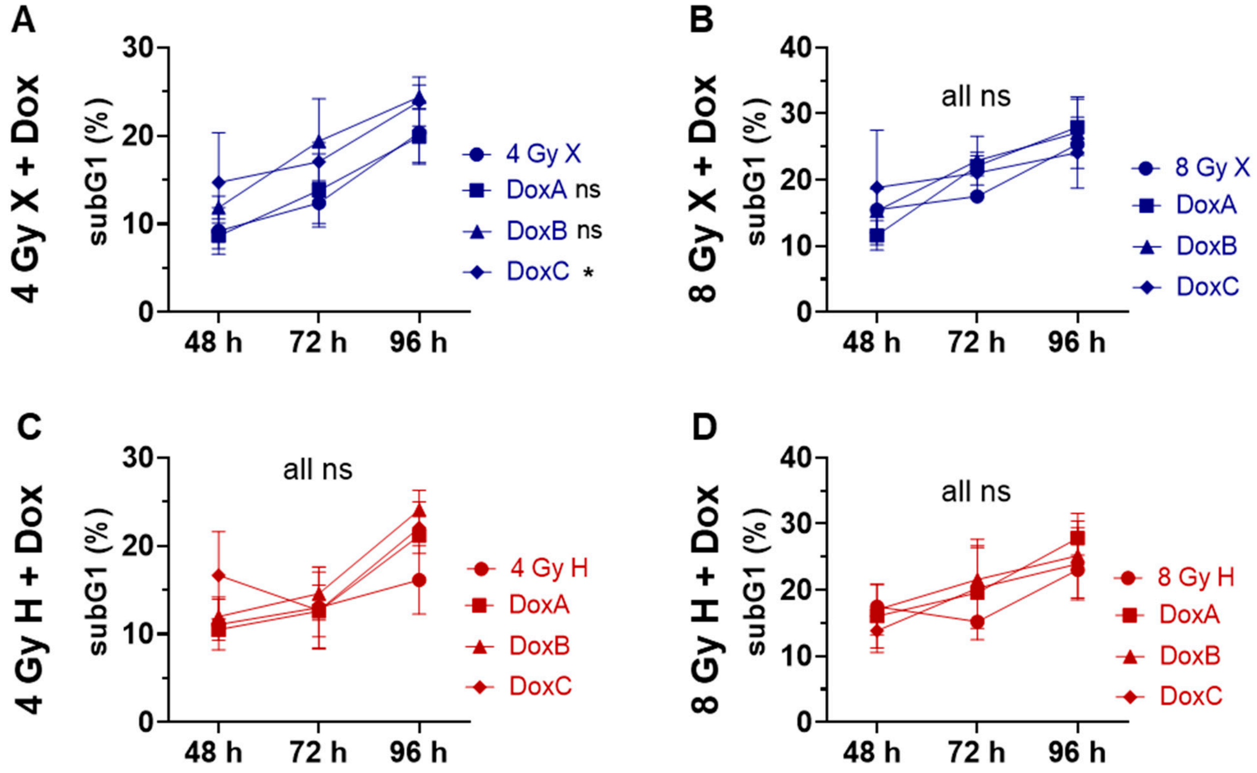

Supplement Figure S1: Flow cytometry analysis of additive effects of combined treatment compared with monotreatment (radiation only) of relative subG1 phase (subG1) of HMEC-1 cells following combined treatment with 10 nM DoxA (pre-IR), DoxB (pre- and post-IR), or DoxC (post-IR) and (A) 4 Gy X (blue), (B) 8 Gy X (blue), (C) 4 Gy H (red), (D) 8 Gy H (red). Dox treatment schedule details in Fig 1 C. $n \geq 3$, statistical analysis: paired t-test for whole curve comparing monotreatment vs. combined treatment. p values: > 0.05 (not significant, ns) and <0.05.

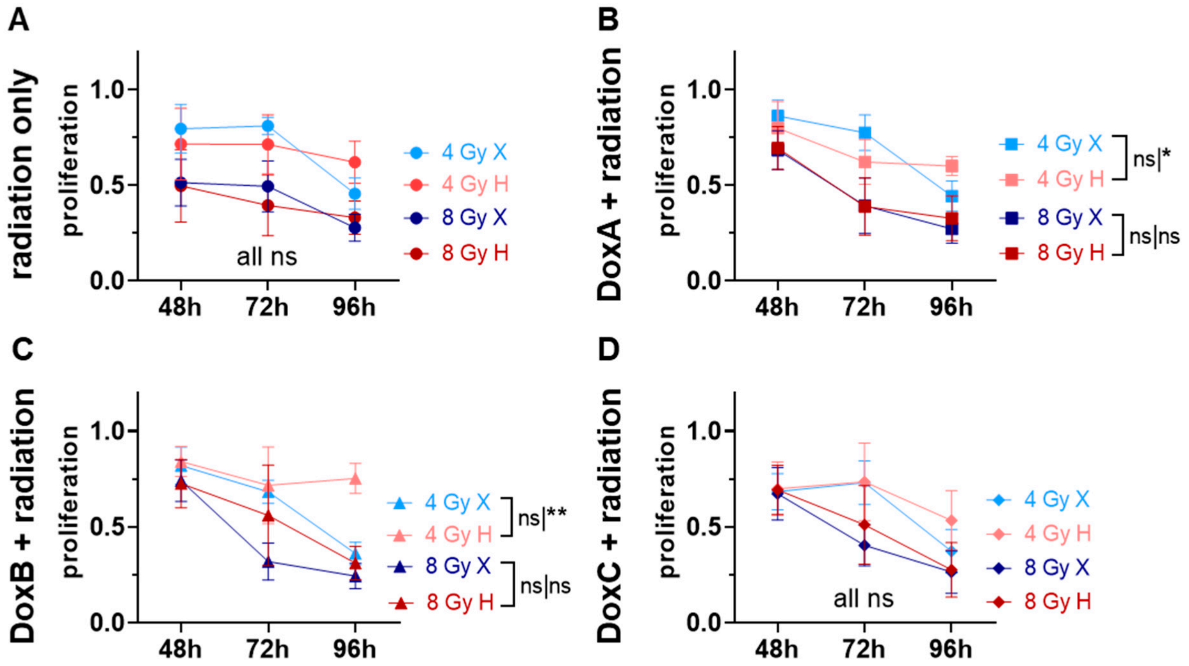

Supplement Figure S2: Proliferation assay comparing relative number of proliferating cells (proliferation; normalized to matched control) X vs. H of HMEC-1 cells following (A) radiation only with 4 and 8 Gy X (light/dark blue) or 4 and 8 Gy H (light/dark red) radiation. (B-D) combined radiation treatment with (B) DoxA (pre-IR), DoxB (pre- and post-IR), or DoxC (post-IR). Dox treatment schedule details in Fig 1 C. $n \geq 3$, statistical analysis: paired t-test for whole curve or unpaired t-test for 96 h time point shown as (whole curve | 96 h) comparing matching X vs. H. *p* values: > 0.05 (not significant, ns), <0.05 (*), and <0.01 (**).

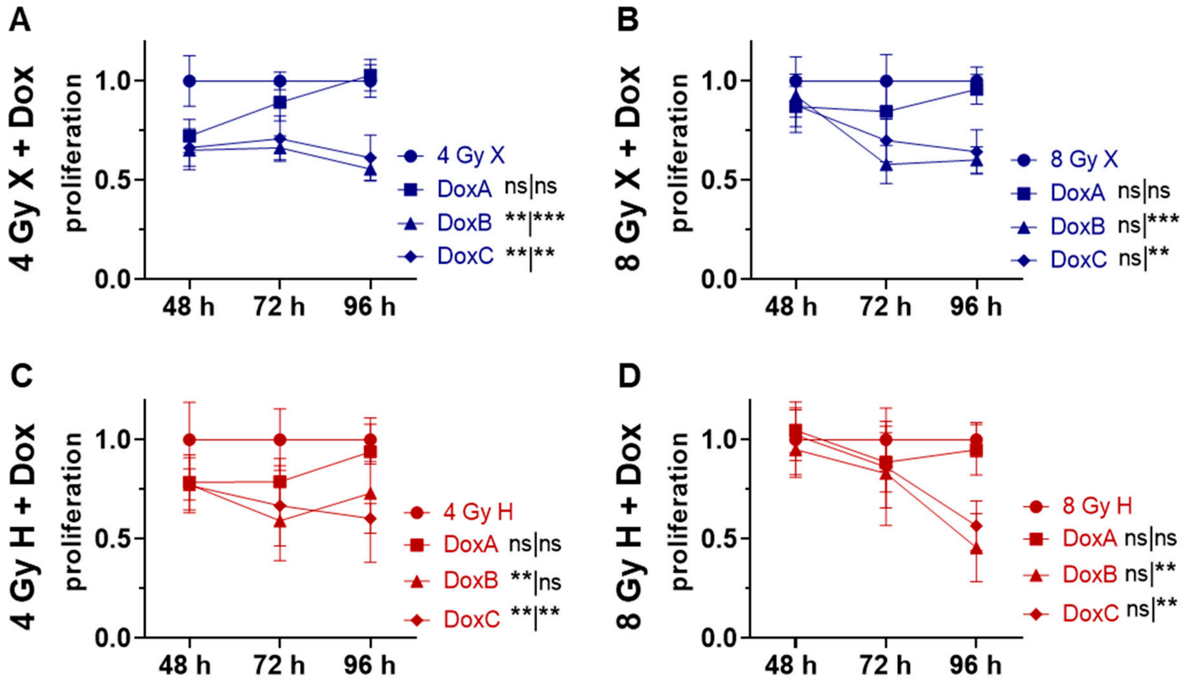

Supplement Figure S3: Proliferation assay analysing the additive effects of combined treatment compared with monotreatment (radiation only) of relative number of proliferating cells (proliferation; normalized to matched control) of HMEC-1 cells following combined treatment with 10 nM DoxA (pre-IR), DoxB (pre- and post-IR), or DoxC (post-IR) and (A) 4 Gy X (blue), (B) 8 Gy X (blue), (C) 4 Gy H (red), and (D) 8 Gy H (red). Dox treatment schedule details in Fig 1 C. $n \geq 3$, statistical analysis: paired t-test for whole curve or unpaired t-test for 96 h time point shown as (whole curve | 96 h) comparing monotreatment vs. combined treatment. p values: > 0.05 (not significant, ns), <0.05 (*), <0.01 (**), and <0.001 (***).

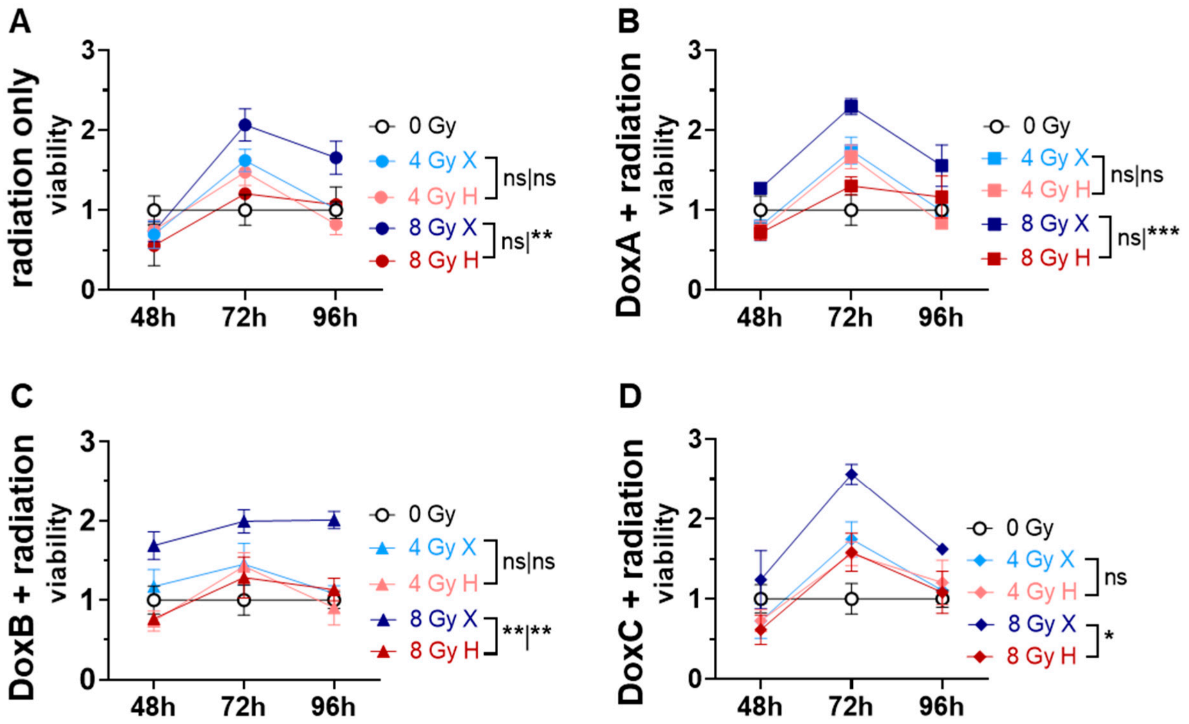

Supplement Figure S4: Cell viability assay comparing relative number of viable cells (viability; normalized to matched control) X vs. H of HMEC-1 cells following (A) radiation only with 4 and 8 Gy X (light/dark blue) or 4 and 8 Gy H (light/dark red) radiation. (B-D) combined treatment with (B) DoxA (pre-IR), DoxB (pre- and post-IR), or DoxC (post-IR). Dox treatment schedule details in Fig 1 C. $n \geq 3$, statistical analysis: paired t-test for whole curve or unpaired t-test for 72 h time point shown as (whole curve | 72 h) comparing matching X vs. H. p values: > 0.05 (not significant, ns), <0.05 (*), <0.01 (**), and <0.001 (***).

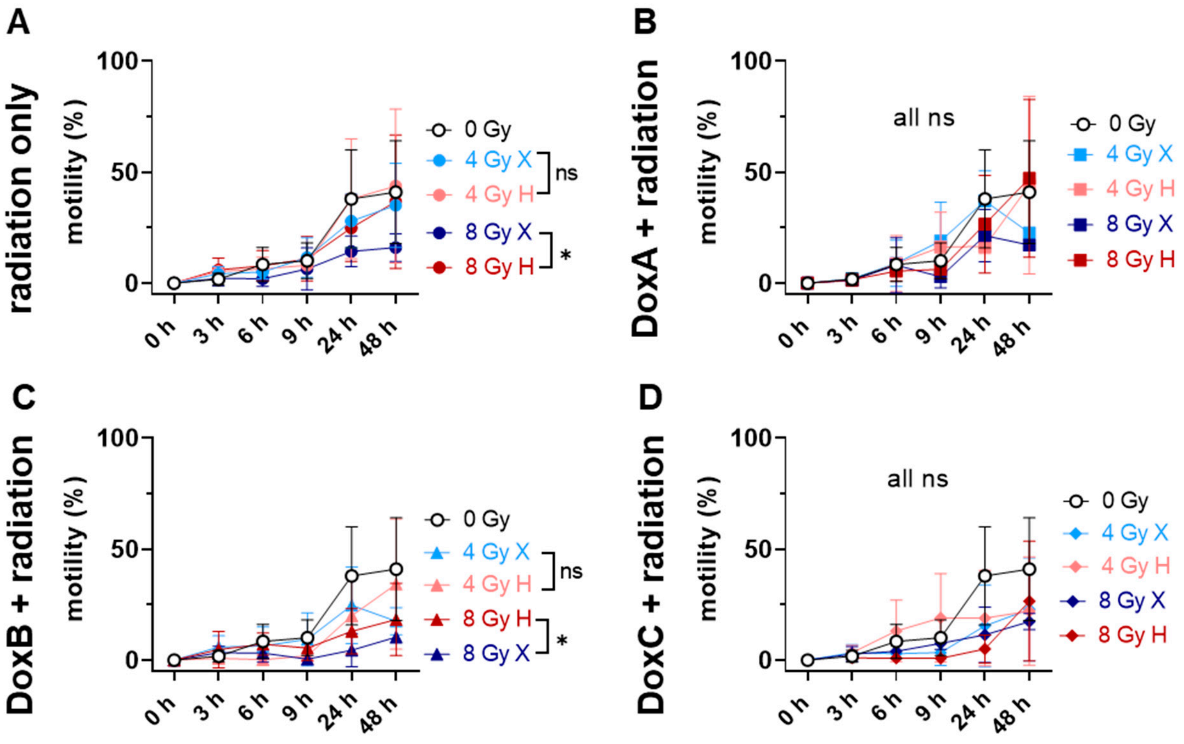

Supplement Figure S5: Migration assay comparing relative number of motility X vs. H of HMEC-1 cells following (A) radiation only with 4 and 8 Gy X (light/dark blue) or 4 and 8 Gy H (light/dark red) radiation. (B-D) combined treatment with DoxA (pre-IR), DoxB (pre- and post-IR), or DoxC (post-IR). Dox treatment schedule details in Fig 1 C. $n \geq 3$, statistical analysis: paired t-test for whole curve comparing X vs. H (3 - 24 h). *p* values: > 0.05 (not significant, ns) and <0.05 (*).

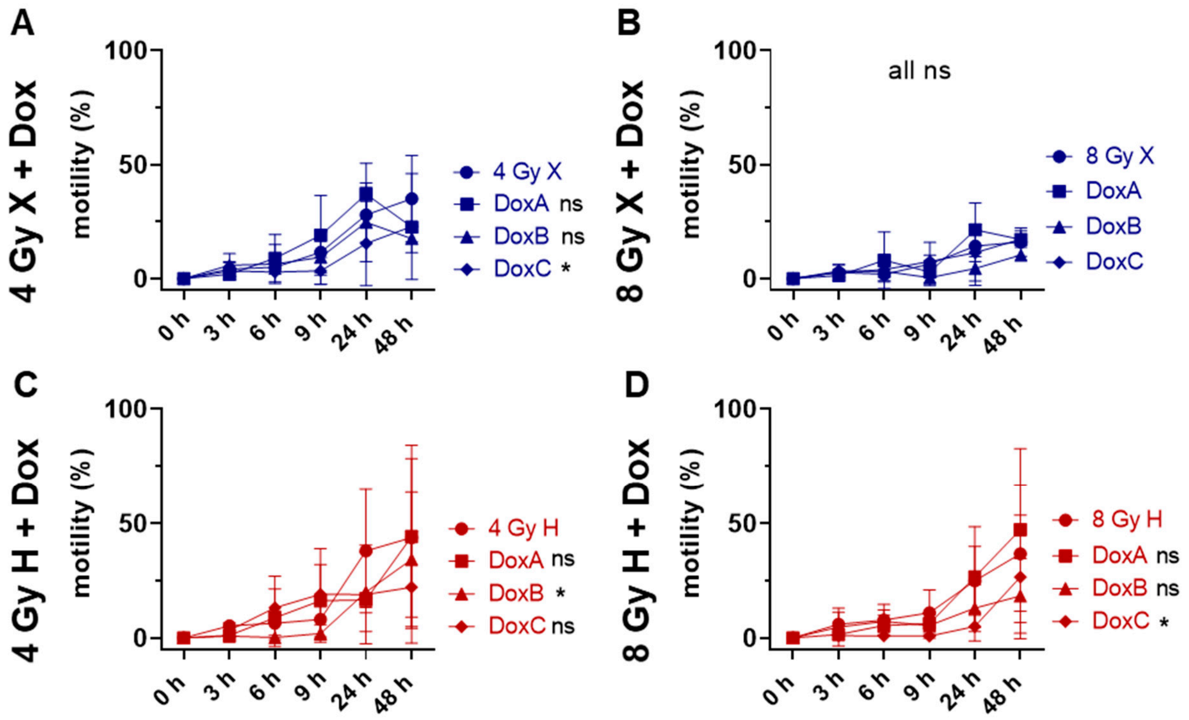

Supplement Figure S6: Migration assay analysing the additive effects of combined treatment compared with monotreatment (radiation only) of motility of HMEC-1 cells following combined treatment with 10 nM DoxA (pre-IR), DoxB (pre- and post-IR), or DoxC (post-IR) and (A) 4 Gy X (blue), (B) 8 Gy X (blue), (C) 4 Gy H (red), and (D) 8 Gy H (red). Dox treatment schedule details in Fig 1 C. $n \geq 3$, statistical analysis: paired t-test for whole curve comparing monotreatment vs. combined treatment (0 – 48 h). *p* values: > 0.05 (not significant, ns) and <0.05 (*).

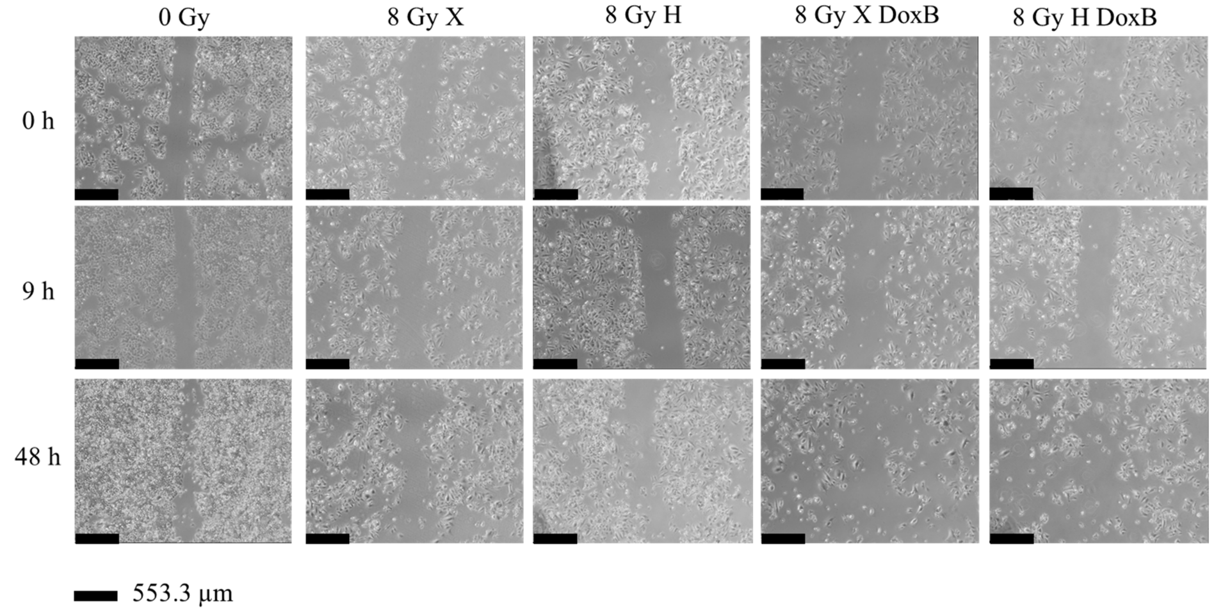

Supplement Figure S7: Exemplary images of migration assay with HMEC-1 cells following 8 Gy X or H radiation and combined treatment with DoxB (pre- and post-IR; details see Figure 1C) at different time points post scratch (0 h, 9 h, 48 h). 4 x magnification and scale bar = 553.3 µm.

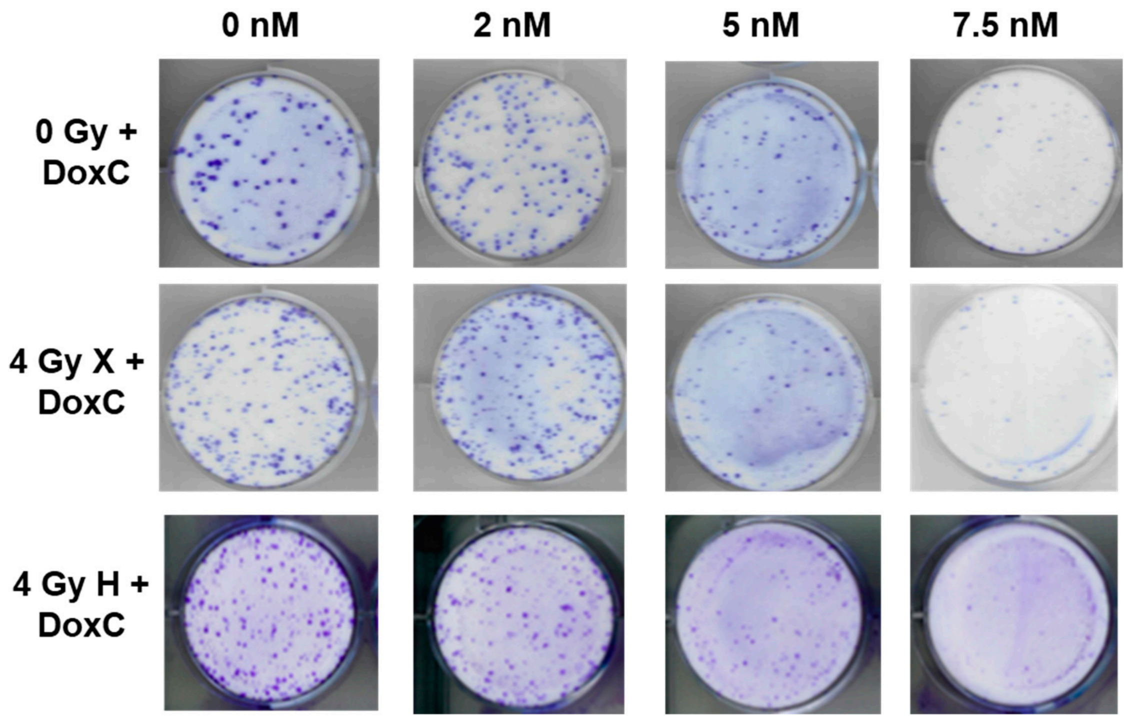

Supplement Figure S8: Exemplary images of colony formation assay with HMEC-1 cells following 4 Gy X or H radiation and combined treatment with DoxC (post-IR; details see Figure 1C).

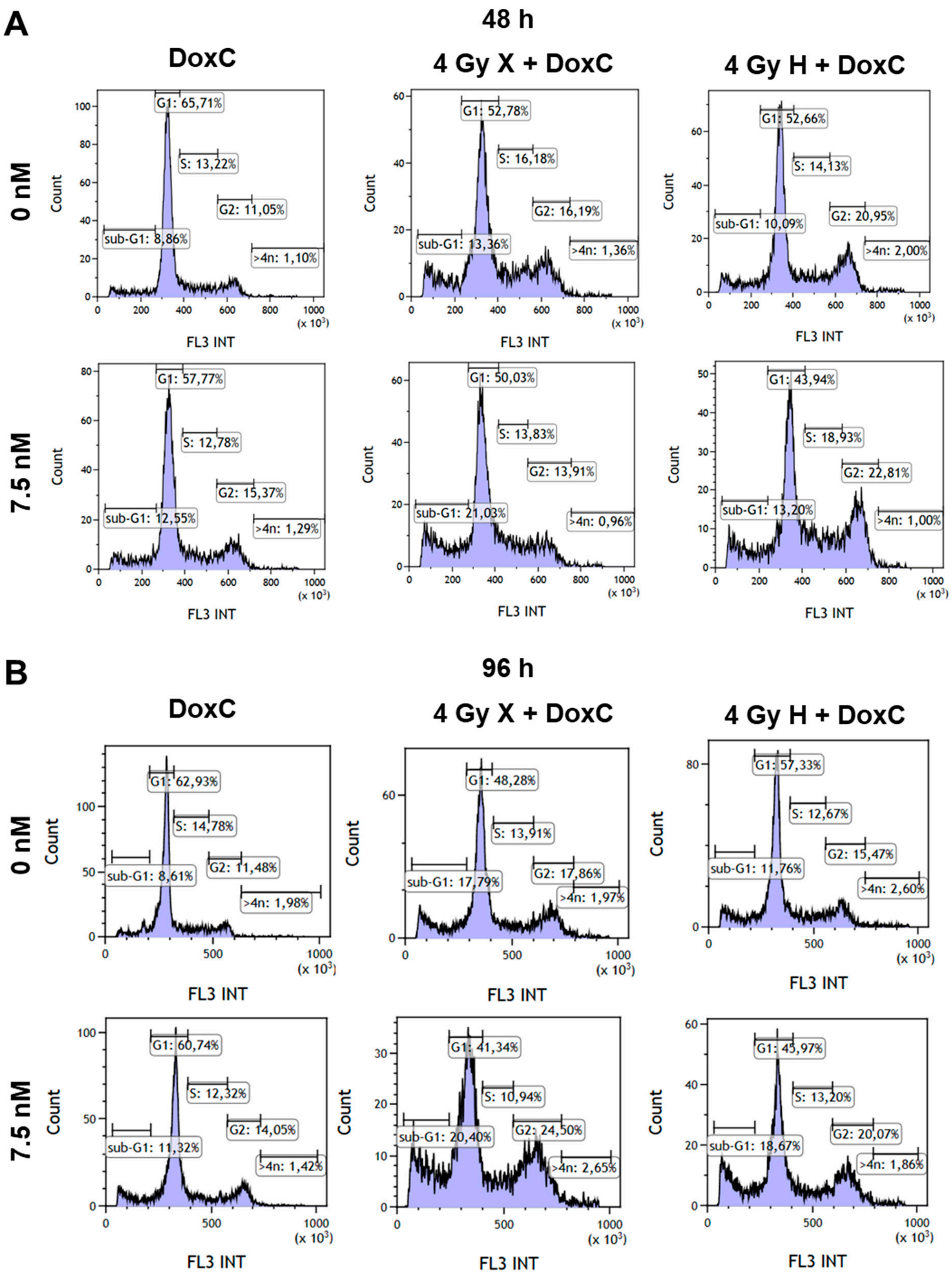

Supplement Figure S9: Exemplary histograms from flow cytometry measurements with HMEC-1 cells following monotreatment with DoxC (post-IR; details see Figure 1C) or combined treatment with 4 Gy X or H radiation (A) at 48 h time point and (B) at 96 h time point.
